# Evaluation of Microencapsulated Synbiotic Preparations Containing Lactobionic Acid

**DOI:** 10.1007/s12010-021-03622-9

**Published:** 2021-07-20

**Authors:** Kamila Goderska, Patryk Kozłowski

**Affiliations:** Department of Food Technology of Plant Origin, Department of Fermentation and Biosynthesis, Faculty of Food Science and Nutrition, Poznan University of Life Sciences, Wojska Polskiego 31, 60-624 Poznan, Poland

**Keywords:** Synbiotic preparations, Microcapsules, Probiotic, Prebiotic, Lactobionic acid

## Abstract

The aim of this paper was to assess the prebiotic properties of lactobionic acid in the human gastrointestinal model. Five different strains of probiotic, or potentially probiotic, bacteria were used in the microencapsulation process; these were *Lactobacillus casei* Shirota, *Lactococcus lactis* ATCC1, *Lactobacillus fermentum*, *Bifidobacterium bifidum* DSM 20456, and *Bifidobacterium bifidum* DSM 20082. Starch with a concentration of 4% (w/v) and a degree of substitution of 0.03 was used to produce the microcapsules. The alginian microcapsules we produced functioned as a protective barrier for the probiotic microorganisms closed in them, protecting them from adverse conditions in the human digestive tract. The microorganisms could thus survive the encapsulation process and the in vitro model digestion process while retaining the ability to produce biomass. Factors such as pH and time affect the solution of alginate microcapsules. The capsule solution began when a pH of 7.4 was reached; this corresponded to pH in the target probiotic site, an in vitro model of the colon. The capsules had completely dissolved after 24 h of digestion at a pH of 8. The addition of lactobionic acid stimulated the growth of probiotic and potentially probiotic bacteria, thus confirming its prebiotic properties.

## Introduction

The beneficial pro-health properties of probiotics and prebiotics are the reason for the development of functional food products and pharmaceutical preparations worldwide. The effectiveness of probiotics in the host organism depends largely on the survival and viability of probiotic bacteria and their release in the intestine in suitable amounts. Various microencapsulation techniques have thus been used to increase the survival of probiotic microorganisms during digestion and their release in the appropriate stage of the digestive tract [1, 2]. It should be borne in mind that the microencapsulation of probiotics does not reduce the survival of cells, their activity, or their ability to adhere to the intestinal mucosa, but only protects the probiotics from the unfavorable environment prevailing in the stomach and small intestine [3, 4].

In creating a synbiotic preparation, prebiotics that show synergy with the probiotic-enabling lactic fermentation should be selected. Lactobionic acid seems to be a good candidate for the prebiotic substance, due to its resistance to digestive enzymes and its low rate of absorption in the small intestine, meaning it can ferment in the further part of the digestive tract through the intestinal microflora that naturally occur there.

Lactobionic acid (LBA) is an aldonic acid with the structural formula C_12_H_22_O_12_, and its systematic name is 4-O-β-D-galactopyranosyl-D-gluconic acid. Its molecule is made up of a galactose molecule combined via an ester bond with a gluconic acid molecule. The molecular weight of lactobionic acid is 358.3 Da [5]. The high number of hydroxyl groups in LBA gives its hygroscopic and gelling properties. A characteristic feature of lactobionic acid is its amphiphilicity—its ability to dissolve in both polar and nonpolar solvents [5].

The substrate for the production of lactobionic acid is lactose, which is converted to LBA through oxidation. There are currently four known reactions that produce lactobionic acid: chemical lactose oxidation, electrochemical lactose oxidation, catalytic lactose oxidation, and microbiological lactose oxidation.

The lactobionic acid available on the market is mainly produced through chemical synthesis, which is responsible for it high price of about €1770 per kilogram of pure LBA. In 2007, the size of the lactobionic acid market was estimated at around 17000 tonnes, with a 5% growth rate for the following years. The largest LBA manufacturers in the world include Solvay and FrieslandCampina Domo [6]. The properties of lactobionic acid have given its broad application in various sectors, which at present include the pharmaceutical and biomedical industries, the food industry, the cosmetic industry, and the chemical industry.

The Japanese company Snow Brand Milk Products has developed a functional milk-based drink with the addition of lactobionic acid, which is aimed at reducing the calcium deficiency inherent in the Japanese diet. In 1994, a study that aimed to determine the organism’s tolerance to lactobionic acid showed that a dose of 24 g/day was tolerated by the digestive system. Lactobionic acid is resistant to digestive enzymes and is poorly absorbed in the small intestine, so it can be used as a substrate for lactic fermentation by the naturally occurring intestinal microflora [5]. In 2011, the US Food and Drug Administration (FDA) approved calcium lactobionate (E-399) as an antioxidant and acidity regulator for foods.

In the pharmaceutical and biomedical industries, lactobionic acid has been used as a biocompatible and biodegradable ligand. Additionally, its chelating properties make it possible to use it as one of the components of the fluid used in organ transport [5, 7]. Preparations that sooth the symptoms of diseases such as atopic dermatitis, rosacea, and seborrheic dermatitis often contain lactobionic acid, as it has weak skin-irritating properties, which is important in the prevention of skin diseases. An increasing number of cosmetics contain lactobionic acid, as it supports the process of exfoliation of the epidermis and stimulates skin cells to synthesize collagen [8].

In the chemical industry, lactobionic acid is used as a surfactant and is included in biodegradable sugar-based surfactants that are less environmentally polluting than traditional surfactants [9]. N-aklomides of lactobionic acid have been added to anticorrosion agents used in the metal industry.

The term prebiotic was introduced in 1995 [10] and refers to food components that are not digested in the gastrointestinal tract but that have a beneficial effect on the body by stimulating the growth or activity of certain microorganisms that colonize the colon, mainly *Bifidobacterium* and *Lactobacillus* [11]. The use of prebiotics [12]:

- Stimulates the growth of certain bacterial strains
- Lowers the pH of the contents of the intestine
- Relieves constipation and prevents diarrhea
- Lowers blood cholesterol levels
- Supports the absorption of minerals such as magnesium, calcium, and iron
- Affects the metabolism of proteins and fats
- Has a positive effect on the immune system and reduces the risk of colon cancer

Prebiotics include indigestible oligosaccharides, such as lactulose, oligosaccharides derived from soya, and fructooligosaccharides. The most important polysaccharides with prebiotic properties are pectin, cellulose, inulin, resistant starch, and hemicellulose [13]. The ideal prebiotic should have such properties as [11]:

- Resistance to the hydrochloric acid, bile salts, and hydrolyzing enzymes in the intestine
- Not being absorbed in the upper part of the digestive tract
- Easily undergoing fermentation under the influence of beneficial intestinal microflora

The term “probiotic” is derived from the Greek words “pro” and “bios,” which mean “for” and “life,” respectively. Currently, the term is used to describe nonpathogenic organisms with beneficial effects on the host organism, and according to the 2002 FAO/WHO definition, probiotics are living microorganisms that, when administered in sufficient quantities, have beneficial health effects [11].

Probiotics are mainly lactic acid bacteria (*Lactobacillus*), which are able to settle in the gastrointestinal tract of humans; however, Bifidobacteria and the yeast *Saccharomyces boulardii* are also used [9, 11]. Commercially available probiotics may contain a single strain or a mixture of two or more. A given strain may have different effects on the human body depending on whether it is used individually or in combination with other probiotic strains, and the benefits of probiotic preparations also vary from one patient group to another. One study from 2011 showed that probiotic preparations containing many probiotic strains are more effective than those containing single bacterial strains [14].

Before a microorganism can be considered a probiotic strain, it must have a documented pro-health effect and must meet a number of requirements that enable it to survive the difficult conditions of the digestive tract and settle in the large intestine [11, 15]. Such a microorganism will have the ability to produce lactic acid by fermenting sugars, will adhere to the epithelium of the large intestine, will not be a pathogen, will not produce toxic compounds, will be resistant to the low pH of the stomach and to the action of digestive enzymes and bile, will multiply easily in the digestive tract, will be a stable strain, will produce large amounts of biomass, will have been isolated from the human body (and specifically from the large intestine), will be an totally or relatively anaerobic strain, and will belong to a specific genus and species.

As mentioned above, it is difficult to determine which species of microorganisms play a major role in providing beneficial health-promoting properties. It has been shown that the microorganisms that are considered probiotic are capable of inhibiting the development of pathogenic microorganisms by producing organic acids, hydrogen peroxide, and bacteriocins. The organic acids they produce by sugar fermentation (lactic, acetic, hypurotic, and citric acid) cause the pH in the large intestine to fall outside the optimal range for pathogens. This organic acid–driven pH reduction is also responsible for reducing the adhesion capacity of pathogens to the colon epithelium. The presence of hydrogen peroxide and bacteriocin, mainly nisin, has an inhibitory effect on gram positive G( +) bacteria. Lactose intolerance is a genetically determined deficiency of β-galactosidase that results in the inability to hydrolyze lactose to glucose and galactose.

When undigested lactose reaches the large intestine, it is broken down by bacterial enzymes, which can lead to osmotic diarrhea [15, 16]. A deficiency of β-galactosidase needs not only to have a genetic basis, as it can also be acquired through damage to the intestinal mucosa or through infection with a rotavirus that infects lactase producing microorganisms. Individuals who are in this way intolerant of milk and milk products experience discomfort in the abdominal cavity and may also suffer from bloating and diarrhea. Probiotic bacteria help to alleviate the symptoms of lactose intolerance, because the microorganisms in probiotic preparations are capable of biosynthesizing β-galactosidase [17].

One of the causes of liver cancer is the consumption of food contaminated with aflatoxin B_1_, which causes characteristic genetic changes in the cancer gene; some strains of probiotic bacteria present in the intestine are able to bind and neutralize aflatoxin B_1_, resulting in less bioabsorption of the toxin from the intestines. By limiting the development of pathogenic microflora that biosynthesize certain fecal enzymes (glycosidase, nitroreductase, and β-glucuronidase), probiotics can prevent colorectal cancer [17]. Numerous animal studies have confirmed the ability of probiotic microorganisms to stimulate the host’s immune system. Useful probiotic bacteria also form a biofilm within the mucous membrane of the large intestine, which can act as a natural barrier against HIV [15, 18].

Probiotic bacteria may also increase the activity of B lymphocytes, which are responsible for the synthesis and activity of immunoglobulin A (IgA), thus enhancing phagocytosis itself. Immunoglobulin A is responsible for the immune mechanism within mucous membranes, including the digestive tract. It is responsible for blocking the adhesion of pathogens to the surface of the intestinal mucosa, for bacterial cell agglutination, and for adsorption of food antigens. Additionally, it can neutralize toxins produced by undesirable microflora [19, 20]. Newborn infants lack intestinal microflora, and its first contact with beneficial microorganisms takes place in the maternal birth canal. This and the mother’s milk are the source of the probiotic microflora that settles in the neonate’s intestine. Recent evidence suggests that early exposure of the neonate to bacteria may have a protective role against allergies by stimulating the immune system through intestinal microflora. The role of probiotic bacteria in allergy is supported by observations of differences between allergy sufferers and healthy children. Children with allergies were characterized by intestinal microflora resembling that of an adult, and their allergies were mainly to food. Such observations may indicate a lack of synergism between the immune system and probiotic microflora through limited contact on the part of the infant with probiotic bacteria. On the other hand, a milder immune response was observed in adults with food allergies to milk using probiotic preparations, which may be related to the abovementioned activity of B lymphocytes [17]. Probiotic bacteria are also responsible for vitamin synthesis, mainly of B vitamins.

Synbiotics refer to food ingredients or food supplements that combine probiotics and prebiotics. Synbiotic products have a beneficial effect on the host, improving the survival and implementation of microbial dietary supplements in the gastrointestinal tract by selective growth stimulation or activation of probiotic bacteria metabolism. For a preparation to be called a synbiotic, it must produce synergy between the prebiotic and the probiotic. The initial reason for combining probiotics with prebiotics was the desire to increase the survival of probiotic microflora as it passed through the upper gastrointestinal tract and to deliver the bacteria more efficiently to the colon. Moreover, it has been found that pH, the presence of organic acids, and moisture affect the viability of the probiotic bacteria in dairy products [11].

The microencapsulation of probiotics is presently a popular topic in research into probiotic survival and stability. Microencapsulation is designed to protect probiotic bacteria from the unfavorable environment of the upper part of the digestive tract and to allow the controlled release of the microflora at the desired stage of digestion. Microencapsulation and the addition of prebiotic substances to probiotic products have been successful in increasing the survival of probiotic bacteria in fermented food [1, 2]. Biopolymers such as alginate, starch, gelatin, carrageenan, whey proteins, gum arabic, gellan, and scanty gum are often used for microencapsulation of bacterial cells [3]. Sodium alginate is often used to encapsulate probiotic microorganisms due to its low cost and biocompatibility, and the ease of forming the gel by binding bivalent ions, while allowing for proper separation in the intestine and release of closed probiotic microorganisms. The experiment of Mandala and Puniya proved that the alginine microcapsules in which *Lactobacillus casei* was closed improved its survival under model conditions of the gastrointestinal tract in vitro and thus did not impede its capacity to adhere to the intestinal epithelium. The disadvantages of alginate are its poor resistance to low pH and its ability to dissolve in the presence of ions or monovalent salts that bind calcium ions [3, 21].

A large number of synbiotic preparations aimed at a range of target groups are available on the Polish market. In addition to formulas targeting adults, there are synbiotics aimed at infants, such as “Coloflor Caserio,” a synbiotic product for infants in drop form. Some products aimed at children come in a range of tastes. The probiotics or potentially probiotic microflora in synbiotic preparations is often in freeze-dried form and enclosed in a gel capsule, though there are also products provided in sachets, to be dissolved in water, or as mentioned in drops intended for infants. Synbiotic preparations containing a single strain are available, as are those with a mixture of probiotic strains, such as “EnteroBiotic,” which contain the yeast *Saccharomyces boulardii*.

Most available preparations include fructooligosaccharides and inulin. None of the synbiotics currently available in Poland has been registered as a medicinal product, and so they cannot be attributed medicinal properties. These preparations are presented as dietary supplements or dietary foods for special medical purposes and are thus not subject to the strict quality control that medicinal products undergo. The Polish Pharmaceutical Society’s 2018 review of oral prebiotics, probiotics, synbiotics, and postbiotics available in Poland draws attention to the lack of unification in registration regulations for new preparations, which results in them having different statuses in different countries, so that the same preparation can be classified as a medicinal product and as a dietary supplement. The review also indicates that most available synbiotic preparations contain doses of prebiotic that are several times smaller than those tested in clinical trials.

The aim of our study was to assess the prebiotic properties of lactobionic acid in the human gastrointestinal model. To this end, we analyzed the survival of selected probiotic and potentially prebiotic microorganisms in the gastrointestinal tract in vitro with lactobionic acid (programming the composition of the capsule so that it constitutes a synbiotic preparation with lactobionic acid) and analyzed the survival of selected probiotic and potentially prebiotic microorganisms in the gastrointestinal tract in vitro with lactobionic acid (LBA) capsules.

## Materials and Methods

### Materials

Five different strains of probiotic or potentially probiotic bacteria were used in the microencapsulation process: *Lactobacillus casei* Shirota, *Lactococcus lactis* ATCC1, *Lactobacillus fermentum* (all from the Department of Fermentation and Biosynthesis, Poznań University of Life Sciences), *Bifidobacterium bifidum* DSM 20456, and *Bifidobacterium bifidum* DSM 20082 (both from the Deutsche Sammlung von Mikroorganismen und Zellkulturen).

MRS medium broth (Oxoid, England) (1.0% peptone, 1.0% beef extract, 0.4% yeast extract, 2.0% glucose, 0.5% sodium acetate trihydrate, 0.1% Tween 80, 0.2% dipotassium hydrogen phosphate, 0.2% triammonium citrate, 0.02% magnesium sulfate heptahydrate, and 0.005% manganese sulfate tetrahydrate) was used to culture the probiotic microorganisms.

The addition of lactobionic acid stimulated the growth of the culture, as was clearly evidenced by the growth curve for each culture with the varying LBA additions (Table 1) [22].

**Table 1:** Lactobionic acid concentration in microcapsules for individual strains of probiotic bacteria

Strain	Lactobionic acid concentration [%]
*Lactobacillus casei* Shirota	1%
*Lactococcus lactis* ATCC1	1%
*Lactobacillus fermentum*	2%
*Bifidobacterium bifidum* DSM 20456	1%
*Bifidobacterium bifidum* DSM 20082	0.5%

^*^0.1% (w/v) = 1 mg/cm^3^ LAB

### Preparation of the Inoculum for Microencapsulation

The probiotic bacterial inoculum was prepared from microorganisms originated from a 100 cm^3^ stationary culture in MRS broth (Oxoid, England) medium. The inoculum was cultured for 48 h at 37 °C under anaerobic conditions. The biomass obtained was centrifuged for 15 min at 5000 rpm (MPW 350R, MPW Medical Instruments, Poland).

### Microencapsulation of Probiotic Bacteria

Microencapsulation was carried out in a microbiological laboratory in sterile conditions using the method described by Goderska [23].

A sterile syringe with 0.8-mm-diameter needle was filled with 4% (w/v) cation starch solution containing 100 mM calcium chloride and bacterial suspension. Eight milliliters of sterile lactobionic acid, at the concentration appropriate for the tested strain, was added. The starch employed was soluble in water at 20 °C and had a substitution degree of 0.04 (Central Potato Industry Laboratory, Luboń, Poland). The starch solution containing the bacteria was added drop by drop to a 0.6% (w/v) type MV solution of sodium alginate (Sigma-Aldrich, St. Louis, USA) from a height of about 40 mm and mixed with a magnetic mixer for 15 min at approximately 700 rpm. The developed capsules were filtered off, washed with sterile bidistilled water, and placed for 15 min in a hardening solution of calcium chloride with a concentration of 1.22% (w/v), stirring continuously. Next, the capsules were again filtered off and washed with bidistilled water. Sterile sodium citrate 3% (w/v) was used to dissolve the ready-made microcapsules (POCH, Gliwice).

### Determination of the Number of Viable Cells per Microcapsule

A sample of 1 g of microcapsules containing probiotic or potentially probiotic microorganisms was placed in 10 cm^3^ of sterile 3% (w/v) sodium citrate solution for 15 min to dissolve the capsules. While the microcapsules were dissolving, a series of decimal dilutions from 10^−1^ to 10^−5^ were prepared. Flooding was used to determine the number of living cells, with 1 cm^3^ of the prepared dilutions being applied to the previously prepared Petri dishes, which were then flooded with liquid medium MRS agar (Oxoid, England). After the plate solidified, it was incubated for 48 h under anaerobic conditions at 37 °C. After the incubation time had lapsed, the number of bacterial colonies in the cultures was counted.

### Analysis of the Microencapsulated Synbiotic Preparations Containing Lactobionic Acid

To determine the survival rate of probiotic and potentially probiotic microorganisms in the microcapsules containing lactobionic acid, we used in a model in vitro gastrointestinal tract consisting of a stomach and small intestine. Digestion of the synbiotic preparations was carried out in a 1-l glass vessel, which was capped with a glass cover with holes for the pH electrode (ERH-111 Nr 11,646), thermometer, and the glass tube for taking samples during digestion. Capillaries passed through the cover, allowing reagents to be dosed. The set-up was placed in a water bath and on a magnetic stirrer (IKA Rct basic).

Under sterile conditions, 14 g of microcapsules containing one of the tested strains of probiotic or potentially probiotic bacteria was weighed into a glass vessel, and 100 ml of sterile MRS broth (Oxoid, Anlia) medium was added. The vessel was inserted into the water bath and the temperature was set to 37 °C.

1. The stomach stage of the mixture was lowered to pH = 2 with 1 M HCl, with the calculated dose being added with a peristaltic pump. After the pH-adjusted solution had been obtained, 2 ml of the sample was taken, and 1 ml of previously prepared pepsin (0.0453 g dissolved in 1 ml 0.1 M HCl (Sigma-Aldrich, St. Louis, USA)) was added to the mixture. Weaving was carried out for 2.5 h on a magnetic stirrer and at 37 °C. A 2 ml sample was then taken.
2. In the small intestine stage, 1 M NaHCO_3_ was dosed into the mixture using a pump until a pH of 7.4 was reached. When the pH reached 6, a volume of 2 ml was taken, and the previously prepared 5 ml pancreatic gut extract (0.01 g pancreatin and 0. 06 g salts of bile acid dissolved in 5 ml 0.1 M NaHCO_3_ (Sigma-Aldrich, St. Louis, USA)) was added within 30 min. As with the stomach stage, the digestion process was carried out at 37 °C on a magnetic stirrer for a total of 2.5 h. After the incubation time, a 2 ml sample was taken, after which 1 M NaHCO_3_ was dosed until the pH reached 8. Once the pH had been normalized, the mixture was left overnight at 37 °C, constantly stirring. The next day, 10 ml of the sample was taken.

### Determination of the Number of Living Cells During In Vitro Digestion

After each stage of the in vitro digestion process, a sample was taken, from which a series of decimal dilutions from 10^−1^ to 10^−3^ were made; the end-of-process sample only was used to make decimal dilutions from 10^−1^ to 10^−4^. Then, 1 cm^3^ of each dilution was added to a Petri dish, and liquid medium MRS agar was poured over it (Oxoid, England). After freezing, the plates were incubated under anaerobic conditions for 48 h at 37 °C. The cultures were then read out, counting the number of microbial colonies.

### Statistical Analysis

The number of live bacteria was counted in triplicate using the plate method. Excel 2000 was used to statistically analyze all the experimental designs using mean descriptive statistics and single-factorial analysis of variance for *P* < 0.05.

## Results

**Fig. 1:**
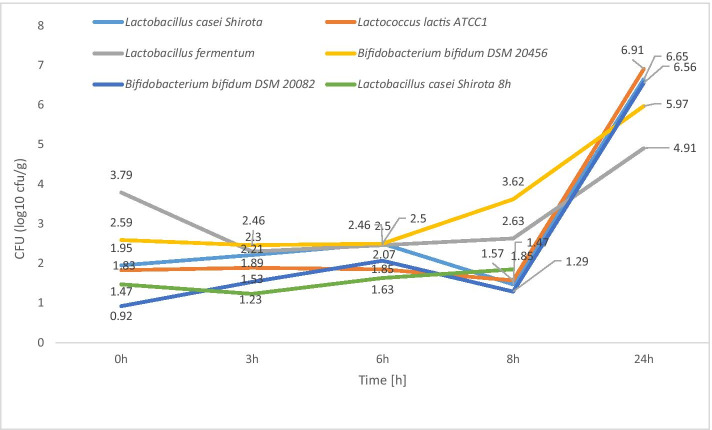
Changes in cell count during in vitro digestion

Time 0 refers to when the pH was stabilized at 2 and in vitro digestion began. The sample for this determination was taken before the addition of the enzyme pepsin. Another sample was taken after the gastric stage (at 3 h in Table 2), after the pH was stabilized to 6, and another sample was taken before the pancreatic-intestinal extract was added to the in vitro model. The 8-h time point in Table 2 refers to when a sample was taken after the small intestine stage, when the pH reached 7.4; the 24-h time point refers to the time at which the sample with all-night digestion at pH 8 was taken. The highest number of probiotic or potentially probiotic microorganisms was recorded after 24 h of the experiment. The highest value of 6.91 log_10_ cfu/g was obtained for *Lactococcus lactis* ATCC1, while the smallest increase was recorded by *Lactobacillus fermentum* (4.91 log_10_ cfu/g). The changes in the number of microorganisms during digestion are shown in Fig. 1.

**Table 2:** Change in cell count during in vitro digestion

Time [h]	*Lactobacillus casei* Shirota	*Lactococcus lactis* ATCC1	*Lactobacillus fermentum*	*Bifidobacterium bifidum* DSM 20,456	*Bifidobacterium bifidum* DSM 20,082		*Lactobacillus casei* Shirota 8 h
CFU [log_10_ cfu/g]							
0	1.95	1.83	3.79	2.59	0.92	1.47	
3	2.21	1.89	2.30	2.46	1.53	1.23	
6	2.50	1.85	2.46	2.50	2.07	1.63	
8	1.47	1.57	2.63	3.62	1.29	1.85	
24	6.65	6.91	4.91	5.97	6.56	no*	

^*^no not determined

Table 3 compares the number of microorganisms enclosed in capsules before digestion with the number of microorganisms contained in capsules after the in vitro digestion.

**Table 3:** Comparison of the number of cells of microorganisms enclosed in microcapsules before and after the digestion process

Strain	Microcapsules [log_10_ cfu/g]	
	Before digestion	After digestion
*Lactobacillus casei* Shirota	4.95	6.65
*Lactococcus lactis* ATCC1	8.07	6.91
*Lactobacillus fermentum*	5.88	4.91
*Bifidobacterium bifidum* DSM 20,456	7.25	5.97
*Bifidobacterium bifidum* DSM 20,082	6.86	6.56

**Fig. 2:**
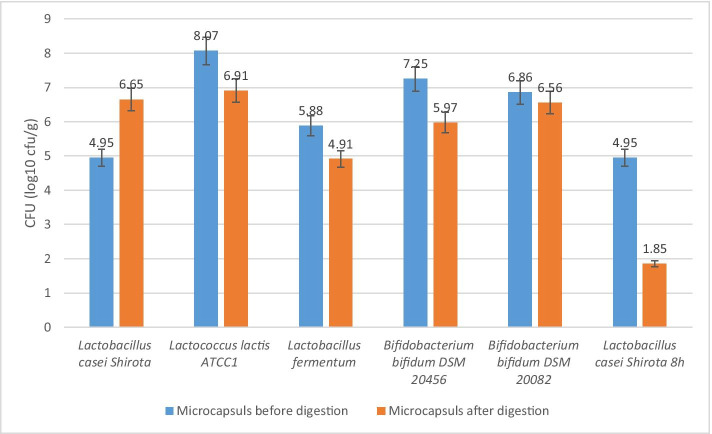
Comparison of the number of cells closed in microcapsules before and after digestion

The greatest number of cells in the microcapsules prior to digestion was found for *Lactococcus lactis* ATCC1 (8.07 log_10_ cfu/g), and the smallest number was 4.59 log_10_ cfu/g for *Lactobacillus casei* Shirota. However, the greatest number of cells in the capsules after digestion—at 6.91 log_10_ cfu/g—was obtained for *Lactococcus lactis* ATCC1, and the smallest number was found for *Lactobacillus fermentum* (4.91 log_10_ cfu/g). A comparison of the number of microorganisms enclosed in capsules is shown in Fig. 2.

The graph shows that *Lactococcus lactis* ATCC1 had the highest number of cells in the microcapsules both before and after digestion process. *Lactobacillus casei* Shirota was the only tested strain to have a higher number of cells in the capsules subjected to the digestion process than in the capsules prior to the digestion process.

## Discussion

An increasing number of synbiotic preparations—combinations of a probiotic with a prebiotic—are becoming available in Poland. Most of these employ fructooligosaccharides or inulin as a prebiotic. Our study has verified the prebiotic properties of lactobionic acid and confirms that it can be used as a prebiotic component in new synbiotic preparations [20]. Our study confirms the literature finding that time and pH can affect the durability of alginine microcapsules. During model in vitro digestion, a gradual dissolution of microcapsules was observed after 8 h, reaching a pH 7.4, which in the model reflects the beginning of the colon in the human digestive system. The capsules were completely dissolved after 24 h of digestion at pH 8 in the model colon—the target probiotic site. In order to confirm the influence of digestion time on the dissolution of alginine microcapsules, capsules containing *Lactobacillus casei* Shirota were prepared, which were digested when a pH of 7.4 was reached. During the digestion of the *Lactobacillus casei* Shirota 8 h liquid sample, a slight turbidity could be observed due to the start of the capsule dissolution process and the whole microcapsules. The presence of probiotic bacteria cells at time 0 (Fig. 1) in the external fluid indicates the beginning of the slow process of alginate capsule dissolution. *Lactobacillus casei* Shirota, *Bifidobacterium bifidum* DSM 20082, and *Lactococcus lactis* ATCC1 at the final stage of the small intestine in vitro model showed a decrease in cell count compared to the other two tested strains (Fig. 1). This may indicate a greater sensitivity to unfavorable conditions at the border of the stomach and the initial fragment of the small intestine. In the final stage of the model in vitro﻿ digestion, all strains showed an increase in the number of cells, which indicates that the probiotic and potentially probiotic microorganisms not only survived under the severe conditions of the human gastrointestinal tract but also preserved their ability to form biomass. Variance analysis of the number of microorganisms cells after the last stage of digestion allows us to conclude that the number of *L. fermentum* cells at the significance level α = 0.05 is statistically different from that of the *L. casei* Shirota, *Lactococcus lactis* ATCC1, and *Bifidobacterium bifidum* DSM 20082 strains. The results shown in Fig. 2 testify to the survival of microorganisms during the encapsulation process (capsules before digestion) and to the protective and permeable alginine properties of the microcapsules used to immobilize the probiotic strains. We statistically compared the average number of cells in the capsules prior to digestion with that in the capsules after digestion, using Student’s *t*-test at a significance level of α = 0.05: the average difference in the number of cells between these groups was statistically insignificant. This test not only confirms that alginate capsules can be used as a carrier for probiotic bacteria, but also shows that lactobionic acid can be used as a prebiotic substance.

Starch with a concentration of 4% (w/v) and a degree of substitution of 0.03 can be used to produce microcapsules. The alginian microcapsules we produced in this way constituted a protective barrier for the probiotic microorganisms enclosed in them, protecting them from adverse conditions in the human digestive tract. The microorganisms survived the encapsulation process and the ﻿in ﻿vitro﻿ model digestion process while retaining the ability to produce biomass. Factors such as pH and time affect the dissolution of alginate microcapsules; this began when a pH of 7.4 was reached, corresponding to the in vitro model of the colon, the target probiotic site. The capsules were completely dissolved after 24 h of digestion at pH 8. The addition of lactobionic acid stimulated the growth of the probiotic and potentially probiotic bacteria, confirming its prebiotic properties. Alginian capsules and lactobionic acid can thus be used to produce new synbiotic preparations.

The study of Dehkordi et al. (2020) aimed to improve the survivability of *L. acidophilus* encapsulated in alginate-whey protein isolate (AL-WPI) biocomposite under simulated gastric juice (SGJ) and simulated intestinal juice (SIJ). Microcapsules were prepared using the emulsification/internal gelation technique. In this study, microencapsulation of *Lactobacillus acidophilus* bacteria was developed into a matrix of AL and WPI based on the various concentrations of AL and WPI and the gum ratio in the aqueous phase. The optimization results indicated that the highest survivability of *L. acidophilus* against SGI was obtained with the incorporation of 4.54% w/v AL, 10% w/v WPI, and 10% v/v gum in the aqueous phase. Physicochemical characterization of the optimal matrix revealed a complete amorphous nature and compact surface with no chemical interactions. Although the proposed biocomposite may be an effective approach for applications in functional foods, for both protected delivery into GI tract and sensorial acceptance, several challenges such as chemical and oil separation need to be overcome before this is used in industry [24].

Considering the size of the market for synbiotic preparations and the numerous clinical studies being conducted on the health impact of probiotic bacteria on the human body, it is worth looking for new methods of immobilization and new carriers for probiotic microflora. The most common prebiotic substances used in synbiotic preparations are inulin and fructooligosaccharides, and use of other prebiotic substances, such as lactobionic acid, is likely to being a number of benefits in terms not only of marketing but also of the raw materials, as even small concentrations of LAB can stimulate beneficial microflora.

## Data Availability

All data generated or analyzed during this study are included in this published article.
